# Genomic adaptive potential to cold environments in the invasive red swamp crayfish

**DOI:** 10.1016/j.isci.2023.107267

**Published:** 2023-07-03

**Authors:** Daiki X. Sato, Yuki Matsuda, Nisikawa Usio, Ryo Funayama, Keiko Nakayama, Takashi Makino

**Affiliations:** 1 Institute for Advanced Academic Research, Chiba University, Chiba 263-8522, Japan; 2 Graduate School of Science, Chiba University, Chiba 263-8522, Japan; 3 Graduate School of Life Sciences, Tohoku University, Aoba-ku, Sendai 980-8578, Japan; 4 Institute of Nature and Environmental Technology, Kanazawa University, Kanazawa 920-1192, Japan; 5 Department of Cell Proliferation, United Center for Advanced Research and Translational Medicine, Graduate School of Medicine, Tohoku University, Aoba-ku, Sendai 980-8575, Japan

**Keywords:** Zoology, Genetics, Evolutionary biology

## Abstract

Biological invasion refers to the introduction, spread, and establishment of non-native species in a novel habitat. The ways in which invasive species successfully colonize new and different environments remain a fundamental topic of research in ecology and evolutionary biology. Here, we investigated the genomic and transcriptomic characteristics of the red swamp crayfish (*Procambarus clarkii*), a widespread invader in freshwater environments. Targeting a recently colonized population in Sapporo, Japan that appears to have acquired a high degree of cold tolerance, RNA-seq analysis revealed differentially expressed genes in response to cold exposure, and those involved in protease inhibitors and cuticle development were considered top candidates. We also found remarkable duplications for these gene families during evolution and their concerted expression patterns, suggesting functional amplification against low temperatures. Our study thus provides clues to the unique genetic characteristics of *P. clarkii*, possibly related to cold adaptation.

## Highlights

• Red swamp crayfish (*Procambarus clarkii*) is a highly invasive species
• Acquisition of cold tolerance is a key to their survival in global habitats
• RNA-seq analysis reveals differentially expressed genes linked to cold exposure
• Remarkable gene duplication may further enhance the cold adaptation mechanism

## Graphical abstract

**Figure:**
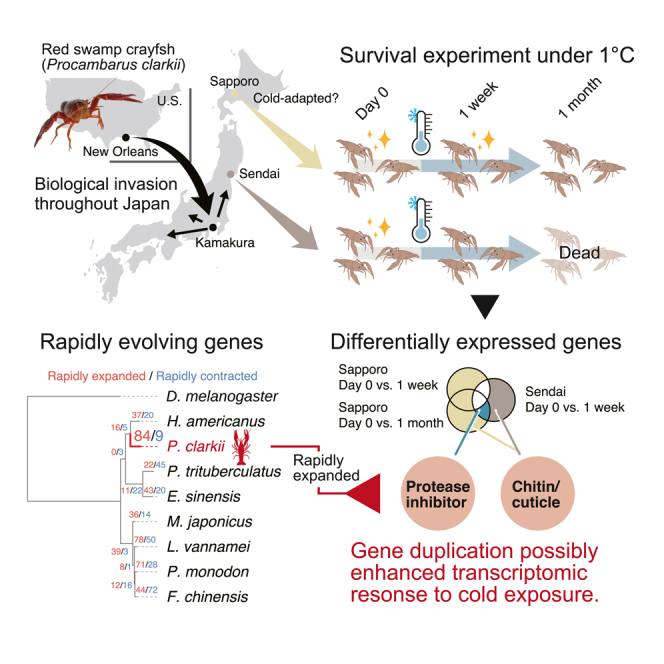

## Introduction

Increasing human activity and improved transport across the globe have facilitated the rapid spread of animals, plants, and pathogens, whether intentional or not. The species that succeed in colonizing new areas can dramatically threaten biodiversity, ecosystem services, and even socioeconomic activities in invaded areas.^1^^,^^2^^,^^3^ Global climate change has an additional impact on biological invasions, often increasing the number and impact of invaders and complicating their management.^4^ Understanding the mechanisms that enable successful invasion processes is therefore an important and urgent objective across a wide range of fields. A long-standing debate in biological invasion concerns the factors that allow invaders to adapt quickly to their new environments, even though they have small population sizes. Several factors, such as propagule pressure, habitat match, and life-history traits, have been proposed to be good predictors of biological invasion success.^5^^,^^6^^,^^7^ A typical genetic signature of invasive species is their relatively high level of genetic diversity despite the reduction in population size.^8^ A recent global study showed that repeated introduction and dispersal along with invasion dilute such demographic influences on genomes, contributing to the maintenance of genetic diversity.^9^ Another genomic signature is the high degree of gene duplication found in invasive invertebrates,^10^ as in other species with a wide range of habitats.^11^^,^^12^ Gene duplication is one of the potential sources of genetic diversity and may contribute to novel protein functions and/or diversified expression patterns. These studies contribute to a growing body of literature on the genomic architecture of invasive species, but specific genetic changes underlying environmental adaptation have not been fully documented.

The red swamp crayfish (*Procambarus clarkii*) is one of the most widespread invertebrate species outside its native range. The native habitat of *P. clarkii* ranges from the southern United States to northeastern Mexico, but it has been transported to all continents except for Australia and Antarctica^13^ and is recognized as one of the most notorious invasive species affecting aquatic biodiversity and ecosystem function.^14^^,^^15^ In Japan, *P. clarkii* was first imported from New Orleans, Louisiana, U.S. in 1927 as feed for American bullfrog aquaculture, and individuals left in the pond spread throughout the Japanese archipelago, becoming one of the most widespread non-native freshwater invertebrates by about the 1950s.^16^ Its invasion success is attributed to its high dispersal ability, omnivorous feeding habits, long lifespan, aggressive behaviors, broad environmental tolerance, and ecosystem engineering activities,^17^^,^^18^ while a key environmental factor that could limit its range is temperature.^19^ In line with the fact that *P. clarkii* inhabits a subtropical climate and has an optimal temperature range of 22°C –30°C,^20^ its habitat in Japan has been limited to warmer areas in Honshu, Shikoku, and Kyusyu. In Hokkaido, the northernmost island of Japan, it has been observed in a limited number of rivers and ponds, where water from hot springs or sewage treatment plants flows in and contributes to high water temperatures throughout the year.^21^ However, some populations, putatively settled by 2008 at the latest,^23^^,^^25^ have been found in the Tonden River in Sapporo City of central Hokkaido, where there is no influx of hot water and water temperatures become extremely low during the winter. Given other recent studies confirming the ability of *P. clarkii* to survive, forage, and reproduce in cold water^22^^,^^24^ and its expanded distribution at high latitudes in North America and Europe,^26^^,^^27^ some populations of this species may have acquired the ability to tolerate cold temperatures. Nevertheless, no studies have so far investigated the detailed genetic mechanism behind the acquisition of cold tolerance. In this study, we aimed to elucidate the genetic mechanism underlying cold adaptation of *P. clarkii* by population genomics, transcriptomics, and comparative genomics approaches.

## Results

### Population history and introduction of *P. clarkii* across Japan

**Figure 1:**
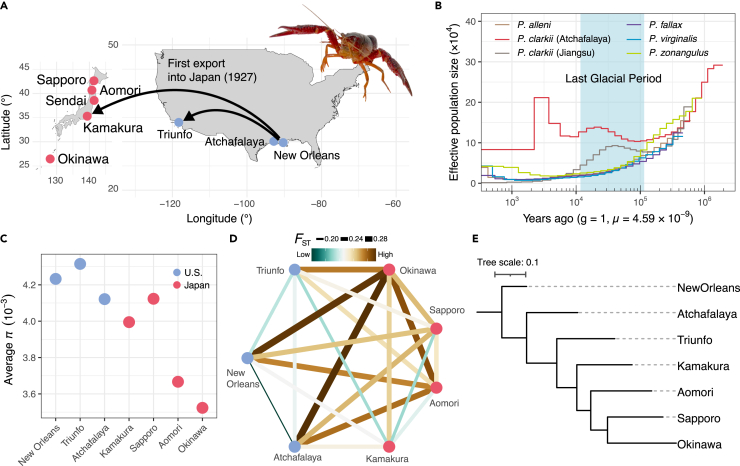
Genomic analysis reveals evolutionary histories and the genetic diversity among *Procambarus clarkii* populations (A) *Procambarus clarkii* samples used in this study. It should be noted that although samples from Sendai were not available for population genetic analyses, they were used for experimental studies. Since their first introduction in Kamakura in 1927, they have expanded their habitat throughout the Japanese archipelago. Photo credit: T.M. (B) Pairwise sequentially Markovian coalescent analysis showed distinct population history of *P. clarkii* compared with the closely related species, which had increased in population size during the last glacial period (shown in the light blue background). (C) Nucleotide diversity among *P. clarkii* populations showed that their genetic diversity decreased along with the expansion of their habitats in Japan, while the Sapporo population showed an increased level of genetic diversity compared with the first introduced population (Kamakura). (D) The genome-averaged *F*_ST_ shows the pattern of genetic divergence between populations. (E) The inferred population phylogeny supports a single origin of introduced Japanese populations.

The crayfish samples used in this study are summarized in Table S1 and Figure 1A. We first performed a pairwise sequentially Markovian coalescent (PSMC) analysis for five closely related species (*P. alleni*, *P. clarkii*, *P. fallax*, *P. virginalis*, and *P. zonangulus*) to investigate the changes in their effective population sizes. We used a *P. clarkii* sample collected from the Atchafalaya Basin in Louisiana and a previously sequenced sample collected in Jiangsu Province, China^28^ for comparison. Given the effects of read coverage on the estimation, we filtered out sites with depth <8 according to previous studies.^29^^,^^30^ The PSMC was performed with 100 bootstrap replicates, and the mutation rate of *Daphnia pulex*, *μ* = 4.59 × 10^−9^,^31^ was used to convert population size to absolute time (as “years ago”) as in a past study.^28^ The result revealed that crayfish decreased overall in population size during the last glacial period (LGP) (Figure 1B), when the northern part of the United States was presumably covered by ice. However, only *P. clarkii* increased in population size during this period, suggesting that its distinct population histories may have been enabled by its genetic and/or ecological potential to adapt to cold climates (Figure 1B). It should be noted that the two *P. clarkii* populations diverged prior to the LGP, and the Jiangsu population has consistently declined in population size since then, in contrast to the estimated trends for the Atchafalaya population, which is stable over time.

Using Pool-seq data of *P. clarkii* from several populations in the U.S. and Japan, we next performed population genetic analysis and compared the level of genetic diversity among habitats. Here, eight samples were pooled for each of the Japanese populations (i.e., Sapporo, Aomori, Kamakura, and Okinawa), while eight, six, and seven samples were pooled for the Atchafalaya, New Orleans, and Triunfo populations, respectively. Populations in the U.S. showed higher nucleotide diversity (*π*) than populations in Japan, and *π* was particularly low in the Aomori and Okinawa populations in Japan (Figure 1C). These results confirmed the effects of repeated population bottlenecks, characterized by a reduction in genetic diversity seen in invading populations. The genetic divergence between the Kamakura and Triunfo populations was lower than that between the Kamakura and the Atchafalaya or New Orleans populations, as indicated by the genome-averaged *F*_ST_ (Figure 1D). The inferred population phylogeny further supported the single origin of *P. clarkii* in Japan (Figure 1E), and the Kamakura population was likely derived from a portion of the native populations that were also introduced in Triunfo^35^ (Figure 1A). Notably, the Sapporo population, although presumably introduced very recently^25^ (Figure 1E), showed a relatively high level of genetic diversity even compared with the Kamakura population.

### Significant cold resistance in Sapporo population

**Figure 2:**
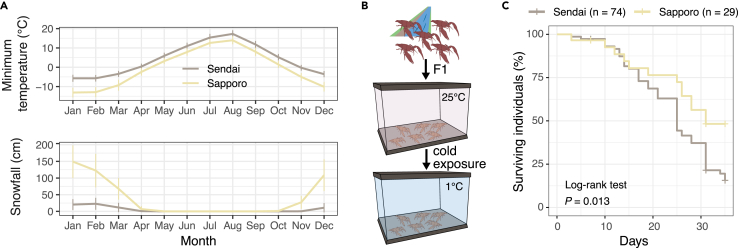
Individuals from the Sapporo population survived longer at low temperatures (A) A comparison of the annual temperature and snowfall of the two regions shows that winter in Sapporo City is characterized by a large amount of snowfall and low temperatures, which can greatly affect crayfish activity and survival. Sixty years of data (1961–2020) were obtained from the Japan Meteorological Agency, and error bars represent SD. (B) Sampling and experimental design of survival analysis. To avoid the plastic response to low temperature, adults collected from the wild populations were kept at 25°C in the laboratory and mated to obtain F1 offspring, and F1 individuals aged 10–20 days were used to compare survival rates at 1°C. (C) The Kaplan–Meier curve of surviving crayfish in cold water. Survival time analysis showed that crayfish from the Sapporo population lived significantly longer than those from Sendai. The experiment was terminated on day 35, and the censoring points including days 7 and 31 are described with “+”. Statistical significance was assessed using the log rank test.

Compared with the native habitat of *P. clarkii*, the Sapporo area has very low winter temperatures (<0°C) (Figure 2A). Furthermore, the city is characterized by high snowfall (Figure 2A), which may affect the activity and survival of crayfish in winter. To investigate their adaptation to cold environments, we next examined the survival rate of *P. clarkii* at low water temperatures and compared it with that of the Sendai and Sapporo populations. The survival experiment was carried out as briefly described in Figure 2B, using animals of the F1 generation reared under the same conditions to study not the plastic but the genetic response to low temperature. Crayfish from the Sapporo population lived significantly longer in cold water than those from the Sendai population (p = 0.013, log-rank test; Figure 2C), which supports the idea that the evolution of cold tolerance may have occurred in the recently introduced crayfish population in Sapporo.

### Candidate genes underlying cold resistance revealed by transcriptome analysis

**Figure 3:**
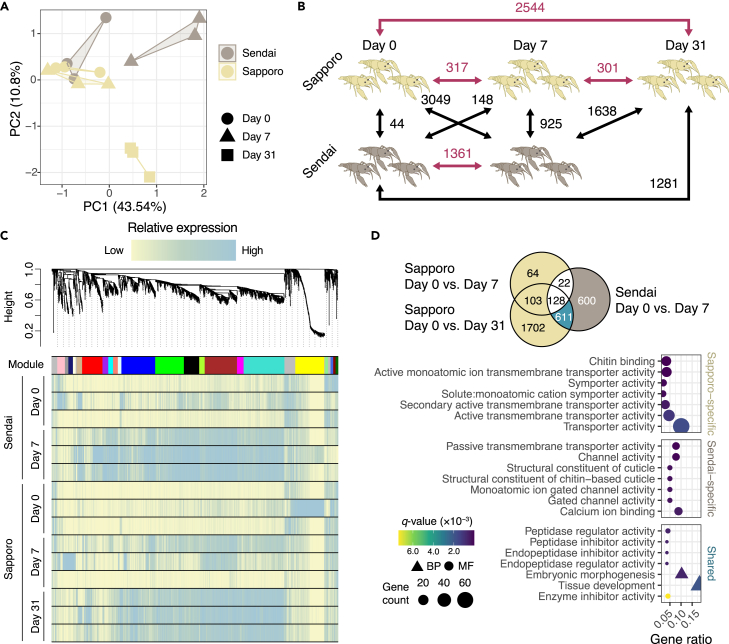
Transcriptomic response involved in the cold resistance of *Procambarus clarkii* (A) Principal component analysis of the gene expression profile showed different transcriptomic responses between the Sapporo and Sendai populations under low-temperature conditions. (B) The number of differentially expressed genes (DEGs) in each comparison. The comparison between conditions with different survival days within a population is highlighted in red, and these within-population DEGs were further investigated in the subsequent analysis. (C) Weighted gene co-expression network analysis revealed several gene clusters (modules) among DEGs. Most genes showed higher expression in long-lived individuals (i.e., day 7 and 31 samples of the Sendai and Sapporo population, respectively), indicating they are expressed in response to cold water temperatures. (D) The top gene ontology (GO) terms significantly enriched in the population-specific or shared DEGs.

To identify the genetic mechanisms that facilitate cold hardiness in *P. clarkii*, we performed RNA sequencing (RNA-seq) and examined the gene expression profiles of the samples used in the survival experiment. Detailed information on the samples and sequenced reads is provided in Table S1. Principal component analysis revealed distinct expression patterns between populations as survival days increased (Figure 3A). While individuals from Sapporo showed no overall change in expression at day 7 compared with day 0, individuals from Sendai showed a significant deviation in expression patterns between days 0 and 7 (Figure 3A), indicating a striking difference in transcriptomic response to low temperature. Differentially expressed genes (DEGs) were then detected between different survival periods in each population, yielding a total of 3,329 DEGs (colored in red in Figure 3B, Supplementary Data 1). Remarkably, few DEGs were detected between the two populations at day 0 (Figure 3B), indicating that their basal levels of gene expression were similar. Meanwhile, hundreds or thousands of genes were found to be differentially expressed under other comparisons, suggesting an acute transcriptomic response to low temperature and its differences between populations. To further characterize the expression patterns in DEGs, we performed a weighted gene co-expression network analysis (Figure 3C) using the list of DEGs. The analysis detected several clusters of genes that are correlated in expression levels, and we found that most DEGs were highly expressed in long-lived individuals (those at day 7 in the Sendai population and day 31 in the Sapporo population; Figure 3C), indicating that the DEGs in the Sapporo population were not as sensitive to the low temperature at day 7 as those in the Sendai population. We then performed gene set enrichment analysis (GSEA) for (1) population-specific DEGs detected between day 0 vs. day 7 or day 31 conditions and (2) population-shared DEGs, which are the intersection of the Sendai DEGs and the Sapporo DEGs found exclusively in the day 0 vs. day 31 condition (Figure 3D), corresponding to the representative expression pattern of the detected DEGs (Figure 3C). Notably, both population-specific DEGs showed a significant overrepresentation (*q*-value <0.05) of genes involved in chitin or cuticle development, whereas the population-shared DEGs represented those involved in peptidase inhibitor activity (Figure 3D, Table S2, Supplementary Data 2–4), which are likely to play a key role in cold hardiness.

### Cold tolerance possibly amplified by significant gene duplication of DEG families

**Figure 4:**
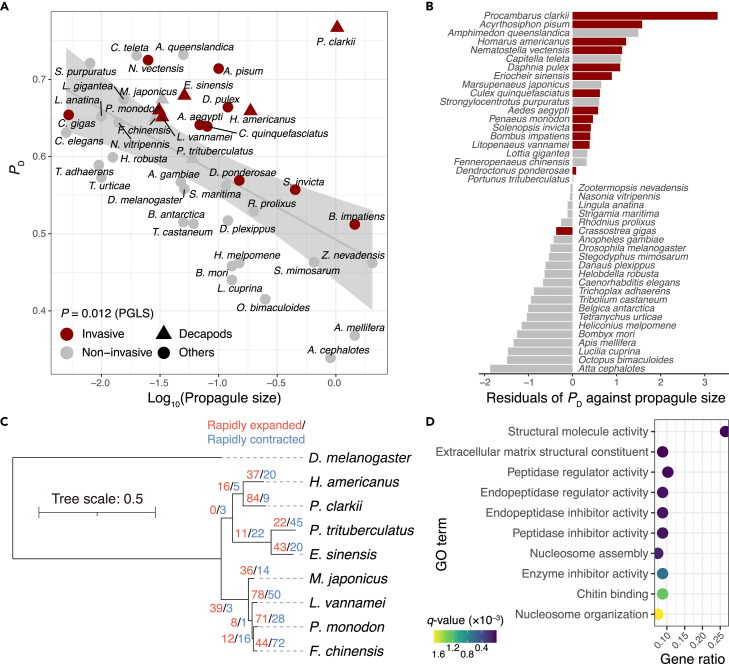
Highly duplicated gene content of *Procambarus clarkii*, represented by rapid evolution of chitin-binding proteins and endopeptidase inhibitors (A) Proportion of duplicated genes (*P*_D_) versus propagule size. A negative correlation was observed between the two indices for whole invertebrates and was tested by phylogenetical generalized least squares. (B) Residuals of *P*_D_ against propagule size calculated for whole invertebrates. (C) Estimated number of gene families that rapidly expanded and contracted in each lineage of decapods. Only the topological relationships between species are shown on the phylogenetic tree. (D) Top GO terms significantly enriched in the 84 rapidly expanded gene families in the *P. clarkii* lineage.

Interestingly, several DEGs were found to be annotated to the same fly genes (Supplementary Data 1). This may indicate that gene duplication plays a critical role in enhancing the transcriptomic response to cold temperatures. Gene expression levels were estimated using only uniquely mapped reads, ensuring that the results were not influenced by multi-mapped reads within the same gene family. Given the potential importance of gene duplication in the adaptability of invertebrates to a wide range of environments,^32^ we next compared the level of gene duplication among species. Seven out of the ten decapod species used in this study were listed in the invasive alien species databases (NEMESIS and/or AquaNIS), and they were likely to have a high proportion of duplicated genes (*P*_D_). *P*. *clarkii* showed a remarkably high *P*_D_ compared with other invertebrates or decapods (Figure 4A and Table S3). We then evaluated the residuals of *P*_D_ against propagule size, as they are known to be negatively correlated^33^ (p = 0.012, phylogenetic generalized least squares). *P*. *clarkii* stood out for this index as well (Figure 4B), indicating that it has a significant level of duplication in its genome, which may be related to its invasiveness.

To further investigate the functional contribution of the duplicated gene content, we estimated the expansion and contraction of gene families across species. Using a phylogenetic tree constructed from the protein sequences of 1,766 single-copy orthologs among nine decapods, CAFE analysis^34^ was performed to estimate the evolution of the 6,517 gene families across species. It should be noted that *P. virginalis* and *Chinoecetes opillo* were excluded from this analysis owing to their incomplete genome annotation (BUSCO <80%) (Table S3), which can lead to an underestimation and/or overestimation of the number of expanded gene families. We identified 93 rapidly evolving gene families at the *P. clarkii* lineage that evolved faster than a mean rate (λ) of gain and loss across all gene families (p ≤ 0.05; Figure 4C). Eighty-four out of the 93 genes were rapidly expanded in the lineage, indicating a remarkably high level of gene duplication, in line with the *P*_D_ index. The GSEA of the genes in these families revealed that those involved in protease inhibitor activity or chitin binding were particularly expanded in *P. clarkii* (Figure 4D), which overlapped with those detected as population-specific or shared DEGs (Table 1). Among them, genes involved in protease inhibitor activity (Spn38F, Spn88Ea, and Tep2) and carbohydrate-binding activity (CG4115 and CG6055) showed a significantly high number of duplication events, which surprisingly coincided with the number of DEGs detected (Table 1). Furthermore, these duplicated gene families showed consistent downregulation/upregulation along with the survival term at a low temperature (Table 1 and Figure S1). This may indicate that the duplication of these genes contributes to the enhanced transcriptomic response to cold stimuli.

**Table 1:** The list of rapidly expanded gene families in *Procambarus clarkii* in which five or more genes were detected as DEGs

Fly gene ID	Fly ortholog	Involved function	Number of DEGs	Number of rapidly expanded genes	Number of genes in genome	DEGs^a^		
						Upregulated	Downregulated	Inconsistent
FBgn0261788	Ank2	Cytoskeletal binding protein	6	12	50	LOC123758779, LOC123763599, LOC123766248	LOC123768127, LOC123768413	LOC123765457
FBgn0038017	CG4115	Carbohydrate-binding activity	12	13	17		LOC123748421, LOC123750641, LOC123752259, LOC123762817, LOC123762818, LOC123764556, LOC123764557, LOC123766926, LOC123771969, LOC123771970, LOC123772041, LOC123772043	
FBgn0031918	CG6055	Carbohydrate-binding activity	15	13	33	LOC123762600, LOC123770700,	LOC123753470, LOC123762822, LOC123766931, LOC123769105, LOC123774340, LOC123774439, LOC123774440, LOC123774441, LOC123774442, LOC123774443, LOC123775203	LOC123762601, LOC123762626
FBgn0013770	Cp1	Degradation of proteins in lysosomes	7	23	38	LOC123752887, LOC123763633, LOC123766918, LOC123768104, LOC123769497, LOC123769501, LOC123769502		
FBgn0053196	dpy	Epidermal-cuticle attachment	8	22	65	LOC123745477, LOC123761630, LOC123762751, LOC123764332, LOC123767307, LOC123767537	LOC123751320, LOC123765839	
FBgn0025874	Meics	DNA-binding transcription factor activity	5	22	56	LOC123746693	LOC123746669, LOC123761190, LOC123762944, LOC123770153	
FBgn0003310	S	Growth regulation	7	9	36	LOC123744904, LOC123745008, LOC123747072, LOC123761759, LOC123769016, LOC123771631, LOC123774785		
FBgn0028986	Spn38F	Serine protease inhibitor	5	5	8	LOC123749022, LOC123750164, LOC123750862, LOC123751037, LOC123752218		
FBgn0028984	Spn88Ea	Serine protease inhibitor	10	7	17	LOC123749015, LOC123749307, LOC123750571, LOC123757200, LOC123757269, LOC123757271, LOC123757272, LOC123757274, LOC123757294, LOC123757299		
FBgn0041182	Tep2	Endopeptidase inhibitor activity	16	10	22	LOC123747531, LOC123748322, LOC123748463, LOC123748498, LOC123749092, LOC123750139, LOC123751068, LOC123752824, LOC123764579, LOC123764584, LOC123764589, LOC123764603, LOC123766600, LOC123766633, LOC123771108, LOC123774736		

a Genes were categorized to those consistently upregulated or downregulated in long-lived individuals, and those with inconsistent expression patterns.

### Signatures of selection driving allele frequency changes in the Sapporo population

**Figure 5:**
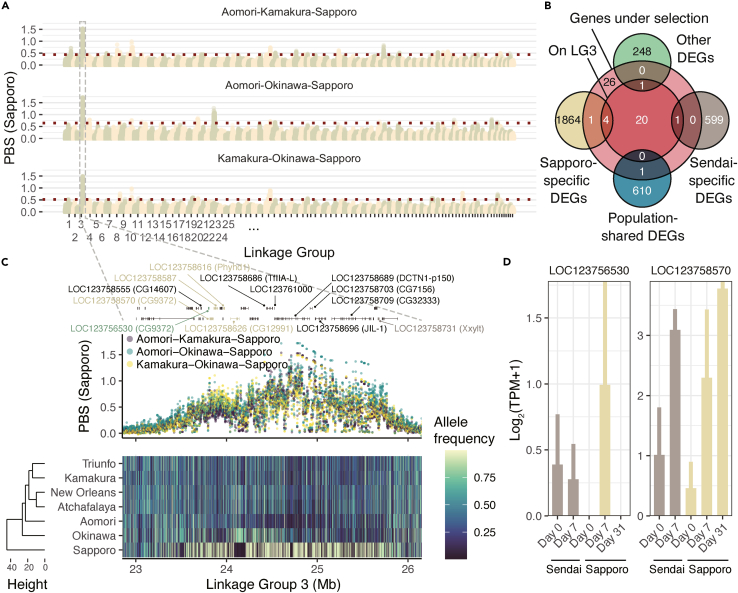
Signature of selection in genomes of the Sapporo population (A) The genomic distribution of the signature of selection as measured by population branch statistics (PBS). Loci above the top 0.1% value of PBS in two or more combinations of populations were considered under selection and used for subsequent analysis. (B) Number of genes detected in the selection scan, intersected with population-specific DEGs, population-shared DEGs, and other DEGs (detected in within-population comparisons). (C) A prominent peak of the selection signature in linkage group 3 showed distinct allele frequency patterns of the Sapporo population. Four Sapporo-specific DEGs (LOC123758570, LOC123758587, LOC123758616, and LOC123758626) and one Sendai-specific DEG (LOC123764393) in addition to another non-specific DEG (LOC123756530) were found under selection in this locus. (D) Gene expression profiles of LOC123756530 and LOC123758570 are shown in log2-transformed TPM. Error bars represent SE.

Finally, we performed the genome-wide scan of the selection signature in the Sapporo population using population branch statistics (PBS),^35^ which was derived from the pairwise *F*_ST_ among Japanese populations (see STAR Methods for details; Figure 5A). We detected 54 genes adjacent to 1,044 loci under selection in the Sapporo population (Supplementary Data 5), of which seven genes were found to be DEGs, indicating that a limited number of DEGs were possibly under selection (Figure 5B). A prominent peak was observed around 23–26 Mb in the linkage group 3 (Figures 5A and 5C), and the hierarchical clustering based on the allele frequencies across the locus showed a distinct frequency pattern of the Sapporo population (Figure 5C). Four Sapporo-specific DEGs (LOC123758570 (CG9372), LOC123758587, LOC123758616 (Phyhd1), and LOC123758626 (CG12991)) and one Sendai-specific DEG (LOC123758731 (Xxylt)), as well as another non-specific DEG (LOC123756530 (CG9372)) were found in this locus (Figure 5C), suggesting that the regulatory elements of these genes may have been under selection. Notably, unlike the other duplicated DEGs, the expression patterns of LOC123756530 and LOC123758570, both predicted to be serine proteases,^36^ are different, with LOC123756530 showing unique downregulation in the day 31 samples of the Sapporo population (Figure 5D).

We further compiled a list of Sapporo-specific non-synonymous substitutions and estimated their effect on the protein function using PROVEAN^37^ and SIFT,^37^ based on the sequence conservation and chemical properties of the substituted residues. Of the 19 non-synonymous variants whose allele frequencies differed significantly in the Sapporo population compared with the other Japanese populations, five substitutions were estimated to be deleterious (i.e., likely to affect protein function) (Tables 2 and S4). Although their functional significance remains to be elucidated, we found one deleterious mutation, F149L, in the LOC123756530 gene.

**Table 2:** Sapporo-specific non-synonymous substitutions located at the significant peak of selection on LG3

Gene ID	Fly ortholog	Position	Ref	Alt	dAF	PROVEAN score	SIFT score
LOC123760978	CG31743	10	V	F	0.51	−0.71	**0.03**
LOC123756530	CG9372	149	F	L	0.65	0.48	**0**
LOC123761000	–	175	L	F	0.88	**−4.00**	–
LOC123758696	JIL-1	227	T	S	0.87	−0.28	**0**
LOC123758717	peng	16	Q	R	0.78	−0.20	**0**

The bold text indicates significant effects of the substitutions as predicted by the PROVEAN score (<–2.5) or SIFT score (<0.05). The Δallele frequencies (dAF) refer to the difference in mean allele frequencies between the Sapporo population and the other three populations in Japan. SIFT could not be run for LOC123761000 owing to a lack of a sufficient number of orthologous sequences.

## Discussion

### Population history of red swamp crayfish and introduction into the Japanese archipelago

Our study illustrates the evolutionary signature associated with the introduction of the invasive red swamp crayfish into the Japanese archipelago. The results of the PSMC analysis suggest a unique history of *P. clarkii*, with population size increasing during the LGP, unlike other related species (Figure 1B). This may reflect this species’ wide range of environmental adaptations, particularly to cold environments, and suggests that its genetic substrates were acquired until 400 kya, when the population expansion gradually began (Figure 1B). We observed this pattern for *P. clarkii* samples collected both in the U.S. (Atchafalaya Basin, Louisiana) and China (Hongze Lake, Jiangsu Province).^38^ However, the two population tracks diverged prior to the LGP, and since the end of the LGP, the Jiangsu population has experienced a reduction in size while the Atchafalaya population has remained stable. These findings suggest that the Jiangsu population likely originated from other northern populations in the U.S. and may have been more susceptible to climatic changes, resulting in significant population bottlenecks. Interestingly, a recent study indicated that the dominant haplotype of the Chinese population (HTCOI01) is not shared with the Louisiana populations.^39^ Thus, it is plausible that native habitats such as the Atchafalaya Basin served as refugia during the LGP, contributing to the persistence of the population.

Japanese populations were estimated to be derived from a single population introduced in Kamakura about 100 years ago (Figure 1E), resulting in reduced genetic diversity compared with native populations (Figure 1C). However, we found a unique genomic signature of the Sapporo population, which has a higher level of genetic diversity than the source population (Kamakura) (Figure 1C). Although we did not detect gene flow from American populations into the Sapporo population, there may be an unstudied ghost population that has influenced the genomic architecture of the population.

### Cold tolerance acquired by genetic changes, reflected in transcriptomic responses

Temperature plays an important role in the geographical distribution of ectotherms, and low temperatures can affect their activity and survival during the winter months. The Sapporo area is characterized by high snowfall and a cold climate in winter (Figure 2A), which would affect the energy metabolism of aquatic organisms like crayfish. Survival experiments using F1 individuals from the Sendai and Sapporo populations revealed the difference in cold tolerance between the two populations (Figure 2C). Although we cannot exclude the possibility of epigenetic inheritance of cold tolerance, our result suggests that genetic changes underlie their phenotypic differences.

The results of the RNA-seq analysis showed that the gene expression patterns of the two groups were indeed different. In the Sendai population, a significant difference was observed between the samples taken on days 0 and 7 (Figure 3A), whereas in the Sapporo population, only the samples taken on day 31 were significantly different from the others. This suggests that the Sendai crayfish, but not the Sapporo crayfish, had already undergone physiological changes by day 7, but these changes may not have been reflected in the survival rate at that time as the survival rate of both populations was high on day 7 (Figure 2C).

### Cold tolerance amplified by significant gene duplication in *P. clarkii* genome

Our analysis revealed that many of the genes differentially expressed in response to low temperature had undergone multiple duplications in the crayfish (Supplementary Data 1). The comparative genomic analysis further revealed a significant level of gene duplication in the *P. clarkii* genome (Figures 4A–4C), which is associated with invasiveness in invertebrates. Notably, rapidly evolving gene families largely overlapped with the DEGs (Table 1 and Figure 4D), strongly supporting the idea that the duplication of chitin- or cuticle-related genes and those involved in endopeptidase inhibitors contribute to the cold tolerance of *P. clarkii* as discussed later. The expression patterns of the duplicated genes were generally similar (Figure S1), suggesting that duplication may help to amplify the function of the genes. While gene duplication generally increases the diversity of gene expression and promotes functional differentiation,^40^^,^^41^ several examples are known where gene duplication amplifies the expression and function of proteins.^42^

The significant level of gene duplication in the *P. clarkii* genome may have been an important precursor for mitigating population decline during the LGP (Figure 1B). The regulatory changes in duplicated genes in response to low temperatures, presumably as occurred in the Sapporo population, may have been adaptive at the time. However, such regulatory changes can be associated with greater metabolic costs^43^^,^^44^^,^^45^ and may be maladaptive at ambient temperatures. Thus, the regulatory changes in response to cold environments may have been lost as temperatures have risen since the end of the Ice Age.

### Genes involved in chitin and cuticular development and difference in cold tolerance among populations

A notable finding in the transcriptomic responses of *P. clarkii* to cold temperature was the upregulation of genes involved in cuticle development in animals that had survived for a long time in a cold environment (Figures 3C and 3D). Significantly, these genes were found as population-specific DEGs in response to low temperature in both the Sendai and Sapporo populations, suggesting that regulatory changes in this biological pathway may underlie the difference in cold tolerance between the populations. Recent transcriptomic studies have reported the upregulation of cuticular and/or chitin-related genes in a wide range of insects,^46^ and related genes may be under selection for cold adaptation.^47^ However, it is unclear how the regulatory changes in these genes affect cold tolerance in arthropods. The increased chitin and cuticle synthesis is likely to be a mechanism to resist desiccation and retain water within the body.^47^ It has also been found that Antarctic arthropods have the ability to avoid freezing or supercooling by altering the melting point of body fluids with dehydration through water-permeable cuticles.^47^^,^^48^ Given that *P. clarkii* in the Sapporo population overwinters in the frozen, snow-covered river, these mechanisms may help them survive in the extremely cold environment. Interestingly, a previous study confirmed the expansion of cuticular gene families in the red swamp crayfish genome,^48^^,^^49^ possibly suggesting the importance of these gene duplications in the evolution of *P. clarkii*.

### Protective effects of protease inhibitors against cold stress

We detected significant duplication and differential expression of Spn38F and Spn88Ea, both serine protease inhibitors (serpins), and Tep2, a thioester-containing protein, in response to low temperature (Table 1). Several studies have documented the upregulation of serpins following freezing exposure in reptiles^50^^,^^51^ or hibernation in small mammals.^51^ In mammals, protease inhibitors are involved in mitigating the adverse effects of protease leakage from lysosomal membrane permeability that occurs during cold stress. Cold exposure caused the disruption of cellular microtubules and other cytoskeletal structures, but pretreatment with protease inhibitors significantly protected cell morphology and function.^52^ The duplicates of Cp1 (cysteine proteinase 1), which is involved in protein degradation in lysosomes, showed correlated expression with serpins (Figure S1), suggesting that regulation of this system may be important for cold adaptation. Hypothermia has been associated with increased production of mitochondrial reactive oxygen species (ROS), and adaptation to low temperature may also be closely linked to the suppression mechanism of ROS.^53^ Given that serpins are also involved in the suppression of ROS, as discussed later, their upregulation may protect proteins and membranes from ROS and oxidative damage.

Another important aspect of these genes is that they regulate the immune system. The thioester-containing protein (Tep) family is one of the pattern-recognition proteins that detect the origin of invading bacteria, which activates the immune system. The invertebrate immune system is composed of cellular and humoral systems. While cellular immunity is involved in phagocytosis, nodule formation, coagulation, and encapsulation of the pathogens, humoral immunity produces antimicrobial peptides and toxic phenolic intermediates or ROS as byproducts of melanization.^54^ The latter system consists of the serine protease (SP) and prophenoloxidase (proPO) activation cascades, which are involved in the activation of Toll signaling or immunodeficiency pathways.^55^ The melanization process must be tightly regulated to prevent excessive production of intermediates and ROS, which can be toxic not only to pathogens but also to the host itself. Serpins act here as the checkpoints to block the SP and proPO cascades. Thus, amplified and/or diversified expression of the duplicated Tep2 and serpin genes in *P. clarkii* may allow precise control of the balance between metabolic and immune stress. Korkut et al. (2018) reported a low mortality rate in signal crayfish (*Pacifastacus leniusculus*) injected with pathogenic bacteria at low temperature (6°C), showing higher phagocytic activity and lower activity of the proPO system and melanization at this temperature.^56^ This result suggests that deactivation of the humoral immune system may mitigate the deleterious effects of intermediates and antimicrobial factors produced and may be an adaptive mechanism against bacterial infection at low temperatures. Compared with Spn88Ea (also known as Spn5), which is known to be a negative regulator of the Toll signaling pathway,^57^^,^^58^ the expression of Spn38F (also known as Spn3) and Tep2 may have antimicrobial effects.^59^ Given that many duplicates of these two genes were comparably expressed during days 0–7 in Sapporo, whereas they were significantly upregulated on day 7 in the Sendai population (Figure S1), the humoral immune system and the generation of noxious factors may be relatively downregulated in the Sapporo population compared with the Sendai population. However, it is difficult to infer complex regulation of the functional interaction of the duplicated genes.

### Signature of selection in the Sapporo population

Genome-wide scans of the selection signature detected a significant peak of PBS located at 23–26 Mb on LG3 (Figure 5A). On closer inspection, several peaks of PBS were present at the locus, suggesting that the target of selection may not be limited to a single gene. In particular, LOC123756530 and LOC123758570, putative homologs annotated to the fly gene CG9372, a family member of SP, were located at the second largest peak around 23.8 Mb. As discussed previously, SP is a target of serpins, and differentiation in the loci may therefore be a key to the differential expression of SP, possibly leading to the differential regulation of serpins or related genes. Interestingly, while LOC123758570 was linearly upregulated in the cold environment, LOC123756530 showed inconsistent expression patterns, with individuals on day 31 of the Sapporo population showing reduced expression compared with those on day 7. This may be an antagonistic mechanism to inhibit the SP cascade at low temperatures acquired in this population. Notably, the non-synonymous mutation F149L of LOC123756530 was found to be dominant in the Sapporo population and was predicted to have functional effects (Table 2), which may be relevant to the unique expression pattern.

We found four other deleterious mutations with significantly different allele frequencies in the Sapporo population (Table 2). CG31743 is an ortholog of human CHST11 (carbohydrate sulfotransferase 11) and is putatively involved in carbohydrate synthesis. JIL-1 is a serine/threonine tandem kinase that regulates gene expression levels by controlling the dimethylation mark of the epigenetic histone encoded by His3.^60^ Peng (penguin) is an ortholog of human PUM3 (pumilio RNA-binding family member 3), which has RNA-binding activity. Although their functional significance remains largely unclear, their interaction with a wide range of genes suggests that they may be involved in regulatory changes in cold-responsive genes.

### Conclusion

The red swamp crayfish was previously thought to be able to inhabit only subtropical and temperate regions, but their presence has been confirmed in subarctic regions in recent years. This has an immense ecological impact on the indigenous communities. Our experimental and bioinformatic approaches revealed evolutionary genetic evidence for the ecological question of how this species has come to thrive in such a wide range of environments worldwide. Significant duplications of genes involved in chitin and cuticular synthesis and the protease inhibitors are likely to amplify the transcriptomic response toward low temperatures. The gene duplication would, at the same time, provide a chance for genes to be sub-functionalized, and different usage of genes may result in divergent populations. The genomic resources presented in this study enhance our understanding of the notable invasive capacity of this species and hold valuable implications for the ecological management of the species.

### Limitations of the study

Deeper sequencing for a larger number of samples will be needed to accurately estimate the genotypes and allele frequencies for each population and understand their complex history of adaptation. Although we found a set of genes and amino acid substitutions possibly under selection and/or involved in cold tolerance, it is difficult to determine their functional roles solely by bioinformatic analyses. Functional studies of candidate genes, such as RNAi and/or genome editing experiments are necessary to better comprehend the detailed molecular mechanism of cold tolerance.

## STAR★Methods

### Key resources table

**Table:** 

REAGENT or RESOURCE	SOURCE	IDENTIFIER
**Chemicals, peptides, and recombinant proteins**		

Genomic-tip kit	Qiagen	10262
Maxwell 16 LEV Plant RNA Kit	Promega	AS1430

**Deposited data**		

RNA sequencing data of *P. clarkii*	This study	SRA: PRJDB15225
Genome sequencing data of *P. clarkii*	This study	SRA: PRJDB5062
Genome sequencing data of *P. alleni*, *P. fallax*, and *P. virginalis*	Gutekunst et al.^60^	SRA: PRJNA356499
Genome sequencing data of *P. clarkii*	Xu et al.^28^	SRA: PRJNA727411
Supplementary data	This study	https://figshare.com/s/22f447f7569e78fd7012

**Experimental models: Organisms/strains**		

Red swamp crayfish, *P. clarkii*	Wild-caught	N/A

**Software and algorithms**		

fastp 0.20.0	Chen et al.^61^	https://github.com/OpenGene/fastp
BWA-MEM2 2.0pre2	Vasimuddin et al.^66^	https://github.com/bwa-mem2/bwa-mem2
SAMtools version 1.17	Li et al.^62^	https://github.com/samtools/samtools/releases/
elPrep version 5.1.3	Herzeel et al. 2021	https://github.com/exascience/elprep
bcftools version 1.10.2	Li et al.^64^	https://github.com/samtools/bcftools/releases/
PMSC version 0.6.5-r67	Li et al.^64^	https://github.com/lh3/psmc
PoPoolation version 1.2.2	Kofler et al.^67^	https://sourceforge.net/projects/popoolation/
PoPoolation2 version 1201	Kofler et al.^67^	https://sourceforge.net/projects/popoolation2/
vcf2phylip v2.0	Ortiz et al.^70^	https://github.com/edgardomortiz/vcf2phylip
HAF-pipe	Tilk et al.^72^	https://github.com/petrov-lab/HAFpipe-line
Provean v1.1.5	Choi et al.^36^	https://www.jcvi.org/research/provean
SIFT	Kumar et al.^37^	https://sift.bii.a-star.edu.sg/www/SIFT_seq_submit2.html
survival version 3.5.5	Therneau et al.^74^	https://cran.r-project.org/web/packages/survival/index.html
fastx-toolkit	The Hannon lab	http://hannonlab.cshl.edu/fastx_toolkit
HISAT2	Pertea et al.^75^	http://daehwankimlab.github.io/hisat2/
StringTie	Pertea et al.^75^	https://ccb.jhu.edu/software/stringtie/
R 4.3.0	R Core Team	https://www.r-project.org
TCC version 1.26.0	Sun et al.^76^	https://bioconductor.org/packages/release/bioc/html/TCC.html
WGCNA version 1.72.1	Langfelder et al.^79^	https://cran.r-project.org/web/packages/WGCNA/index.html
clusterProfiler version 4.4.4	Wu et al. 2021	https://bioconductor.org/packages/release/bioc/html/clusterProfiler.html
BLAST v.2.11.0+	Camacho et al.^80^	https://ftp.ncbi.nlm.nih.gov/blast/executables/blast+/LATEST/
CAFE v5.0	Mendes et al. 2020	https://github.com/hahnlab/CAFE5
MAFFT version 7.520	Katoh et al.^82^	https://mafft.cbrc.jp/alignment/software/
trimAl	Capella-Gutiérrez et al.^83^	http://trimal.cgenomics.org/trimal
IQTREE v2.1.1	Minh et al. 2020	http://www.iqtree.org
ape version 5.7.1	Paradis et al.^84^	https://cran.r-project.org/web/packages/ape/index.html
TimeTree	Kumar et al.^85^	http://www.timetree.org
iTOL v5.6.3	Letunic et al.^71^	https://itol.embl.de
Original codes	This study	https://github.com/daikisato12/Sato2023_crayfish

### Resource availability

#### Lead contact

Further information and requests for resources used in this study should be directed to and will be fulfilled by the lead contact, Takashi Makino (tamakino@tohoku.ac.jp).

#### Materials availability

This study did not generate new unique reagents.

### Experimental model and subject details

#### Crayfish samples for population genetic studies

The samples of *P. zonangulus* from the Atchafalaya Basin, Louisiana; two native U.S. populations of *P. clarkii* from the Atchafalaya Basin and New Orleans, Louisiana; and a domestically invaded population of *P. clarkii* from Triunfo Canyon, Santa Monica Mountains^35^ were obtained by Drs. Allison Quan, Katherine M. Pease, and Robert P. Romaire. From 2012 to 2013, Japanese populations of *P. clarkii* were collected from a waterway upstream of the Tonden River (Sapporo City), a waterway in Sannai Maruyama (Aomori City), a waterway around Kamakura Center Park (Kamakura City), and a pond in Kuwae Park (Nakagami District, Okinawa). Although several rivers in Sapporo City have an inflow of warm treated wastewater from nearby plants,^25^ we confirmed that there is no inflow of warm water into the Tonden River during winter (personal communication with a local administrator). Crayfish were maintained at 25°C in the laboratory, and adult samples were sequenced. We did not determine the sex of the samples as it was not necessary for pool-seq and individual sequencing.

#### Crayfish samples for experimental and transcriptomic studies

For the survival experiment and transcriptomic analysis, we collected *P. clarkii* from a waterway upstream of the Tonden River (Sapporo City) and a waterway in Ayashi (Sendai City). Adults collected from the wild populations were maintained at 25°C in the laboratory and mated to obtain F1 offspring. Experiments were conducted using healthy F1 individuals (10-20 mm in size) of unknown sex, as it was challenging to distinguish sexes at the early stage of development.

### Method details

#### Genomic DNA library construction and sequencing

Genomic DNA was extracted from the muscle tissue using a Genomic-tip kit (Qiagen, Hilden, Germany). Paired-end libraries were clonally amplified on a flow cell and sequenced on the Illumina HiSeq 2000 (101-mer paired-end) platform. Individual samples of *P. clarkii* and *P. zonangulus* were re-sequenced for population demographic inference. For pool sequencing (Pool-Seq), eight samples were pooled for each of the Japanese populations (i.e., Sapporo, Aomori, Kamakura, and Okinawa), while eight, six, and seven samples were pooled for the Atchafalaya, New Orleans, and Triunfo populations, respectively. To estimate past population sizes of closely related species, we also obtained individually sequenced reads of *P. alleni*, *P. fallax*, and *P. virginalis* from the NCBI Sequence Read Archive, following a previous study.^61^ We also used the sequence data of a *P. clarkii* sample collected from Hongze Lake, Jiangsu Province, China^62^ for comparison. The information for sequenced reads for these samples are summarized in Table S1.

#### Read mapping and variant calling for pool-seq and re-sequenced data

Paired-end short reads from each pool or individual were first filtered and trimmed by fastp version 0.20.0^63^ with the argument “-q 30 -u 30 -n 10 -l 20 -t 1 -T 1”. The filtered reads were then mapped to the *P. clarkii* genome assembly^64^ by BWA-MEM2 2.0pre2,^66^ sorted by SAMtools version 1.10,^60^ and then processed with elPrep version 5.1.3,^65^ which removed duplicate reads or reads with mapping quality less than 20. The filtered bam files were then used to identify genomic variants using the mpileup command implemented in SAMtools with the arguments “-C 50 -q 20 -Q 20 -d 200”.

#### Estimation of past effective population sizes

We performed pairwise sequentially Markovian coalescent (PSMC)^31^ analysis to estimate historical changes in the effective population size of the five crayfish species (*P. alleni*, *P. clarkii*, *P. fallax*, *P. virginalis*, and *P. zonangulus*). In addition to samples of *P. clarkii* and *P. zonangulus* collected from Atchafalaya Basin, Louisiana in this study, we used publicly available sequences of *P. clarkii* collected from Jiangsu Province, China:^28^ *P. alleni* and *P. fallax* collected from an aquarium; and *P. virginalis* collected in Madagascar.^65^ The mapped reads were used to call single nucleotide polymorphisms (SNPs), and consensus fasta files were generated for each species using bcftools version 1.10.2.^66^ Given the effects of read coverage on the pattern of SNPs, we applied a filtering of site depth ≥ 8 in accordance with previous studies67. The PMSC program version 0.6.5-r67 was then run with the option “-N25 -t15 -r5 -p 4+25∗2+4+6” with 100 bootstrap replicates, and the mutation rate of *Daphnia pulex*, *μ* = 4.59 × 10^−9^,^68^ was used to convert population size to absolute time (as “years ago”) as the previous study.^69^

#### Population genomic analyses

To investigate the genomic signature of population bottlenecks, we used PoPoolation version 1.2.2^67^ to assess and compare the genome-averaged nucleotide diversity (*π*) among *P. clarkii* populations. To avoid the effects of sequencing errors, we first subsampled the mpileup files for each population to obtain uniform coverage according to the protocol described in a previous study.^68^ Briefly, the mode of coverage for a given pool was targeted, and sites with coverage higher than the mode plus half the mode were excluded using the subsample-pileup.pl script. Calculation of *π* was performed with the variance-sliding.pl script for non-overlapping 5-kbp windows across the genome with filtering of coverage and minor allele frequencies.

We also computed the fixation index (*F*_ST_) for each pair of populations using PoPoolation2 version 1201.^69^ The indel-filtered mpileup file containing seven pools was first converted to sync format using the implemented script mpileup2sync.jar. The *F*_ST_ was then calculated for non-overlapping 5-kbp windows across the genome using the fst-sliding.pl script, and the genome-averaged *F*_ST_ was compared among populations. The target minimum and maximum coverage was set at 4 and 16, respectively, given the coverage of the sequenced pools. Based on the vcf file, we further prepared a phylogenetic tree using vcf2phylip v2.0^70^ and iTOL v5.6.3.^71^

#### Detecting the signature of selection

To detect signatures of selection potentially acting in a population, we calculated population branch statistics (PBS)^35^ and performed a genome-wide scan for selection signatures specific to the Sapporo population. The PBS is calculated by comparing the pairwise *F*_ST_ between three samples and is known to be effective in detecting recent selection.^36^ Before calculating the PBS, we estimated the allele frequencies (AFs) of each SNP with high accuracy using HAF-pipe,^72^ which uses haplotype information to infer AFs. The estimated AFs were then used to calculate pairwise *F*_ST_ between populations in 5-kbp windows with 1-kbp overlap across the genome using the standard equation,^73^ followed by the calculation of PBS according to a previous study^74^ using in-house Perl scripts. Analysis was performed only on genomic loci mapped to linkage groups. Loci under selection were defined as those with a PBS value above the top 0.1% compared with the null distribution across the genome. Those showing the signature of selection in the Sapporo population in two or more combinations of Japanese populations (i.e., Aomori–Kamakura–Sapporo, Aomori–Okinawa–Sapporo, and Kamakura–Okinawa–Sapporo), which included 1,044 loci (0.062 % of loci analyzed), were extracted. We associated genes located +/– 50-kbp from the detected loci and defined them as those under selection.

#### Estimation of intolerance of non-synonymous substitutions

Non-synonymous substitutions in genes can affect protein function and may be under selection. To investigate the possible role of non-synonymous mutations introduced in the Sapporo populations, we first compiled a list of non-synonymous substitutions that differ in AFs among populations located within the region under selection. PROVEAN v1.1.5^75^ and SIFT^76^ were then used to estimate the intolerance for these non-synonymous substitutions, based on the evolutionary conservation and the chemical properties of the exchanged residues. Calculations for SIFT were performed on the web server (https://sift.bii.a-star.edu.sg/www/SIFT_seq_submit2.html; last accessed January 2023). A mutation was predicted to be deleterious (i.e., likely to affect its protein function) if the PROVEAN score was less than −2.5 or the SIFT score was less than 0.05.

#### Survival experiment under cold water

To investigate whether *P. clarkii* from Sapporo has a high tolerance to cold water, we compared survival days under low-temperature conditions between the Sendai and Sapporo populations. The sampling and experimental scheme is briefly shown in Figure 2B. To rule out the possibility of a plastic (non-evolutionary) response to low temperature obtained in the wild environment, adults collected from the wild populations were maintained at 25°C in the laboratory and mated to obtain F1 offspring. The F1 individuals (10–20 mm in size) from the Sapporo (n = 29) and Sendai (n = 74) populations were exposed to a low temperature (1°C) for 35 days. The number of surviving individuals was counted during the test. Survival curves of two populations were compared, and statistical significance was assessed via the log-rank test using the survival package^74^ in R.

#### RNA sequencing and differential expression analysis

We performed RNA sequencing (RNA-Seq) analysis to investigate the difference in gene expression profiles following exposure to low temperature between Sendai and Sapporo populations. Total RNA was extracted from 10–20-day-old F1 individuals exposed to low temperature (1°C) for 0, 7, and 31 days. The 31-day Sendai samples are missing because we incidentally failed to extract RNA from them. Tissues were homogenized in BioMasher Standard (TaKaRa, Shiga, Japan), and total RNA was isolated using a Maxwell 16 LEV Plant RNA Kit (Promega, Madison, WI). RNA concentration and purity were assessed using a Nano-Drop® ND-2000 spectrophotometer (Thermo Scientific, Waltham, MA), and total RNA integrity was quantified using an Agilent 2100 Bioanalyzer (Agilent Technologies, Santa Clara, CA). The 15 cDNA libraries were prepared, and 100-bp paired-end reads were sequenced on an Illumina HiSeq 4000 platform at the Beijing Genomics Institute (Hong Kong, China). A total of 193,032,321 raw sequencing reads were generated (10–17 million read pairs per sample; Table S1) and used for gene expression profiling.

Raw reads were checked for quality, trimmed, and filtered using the fastx-toolkit (http://hannonlab.cshl.edu/fastx_toolkit/) with the argument “fastq_quality_filter –v -Q 33 -q 30 -p 50”. The HISAT2 and StringTie pipeline^75^ was then used to map reads, quantify the expression levels for each transcript, and perform differential expression analysis. Here we used uniquely mapped reads to avoid overestimating the expression levels of duplicated genes. We retained genes > 1 transcript per million (TPM) for two or more samples, leaving a total of 19,649 genes for downstream analyses. The overall expression pattern among samples was then evaluated by principal component analysis. Differential expression analysis was performed using the TCC package version 1.26.0^76^ in R to compare the expression levels between samples at different survival days (i.e., 0, 7, 31 days at 1°C) within and between populations.

#### Enrichment and weighted gene co-expression network analysis

To identify biological pathways enriched in detected genes of interest, we performed GSEA for gene ontology (GO) terms and KEGG and Reactome pathways using the clusterProfiler package^77^ in R. The translated sequences of crayfish genes were blasted against those of *Drosophila melanogaster*, and the best-scoring genes were determined as orthologs. If multiple genes of fruit flies had the same score for a given crayfish gene, the one with the longest CDS was chosen as the representative. Fruit fly protein sequences and annotations (BDGP6.32) were obtained from the Ensembl database, release 108.^78^

Weighted gene co-expression network analysis (WGCNA) was performed to characterize the expression patterns of DEGs using the WGCNA package^79^ in R. A total of 3,329 within-population DEGs were used to calculate correlations in expression levels among samples, and co-expressing modules were detected.

#### Estimation of the number of duplicated genes

To investigate the effect of gene duplication on cold tolerance in *P. clarkii*, we estimated the number of duplicated genes in the genomes of related species. In addition to *P. clarkii*, the genomic information of the American lobster (*Homarus americanus*), Chinese white shrimp (*Fenneropenaeus chinensis*), Kuruma shrimp (*Marsupenaeus japonicus*), giant tiger prawn (*Penaeus monodon*), whiteleg shrimp (*Litopenaeus vannamei*), and gazami crab (*Portunus trituberculatus*) were obtained from Ensembl. The genomic information of the Chinese white shrimp (*Fenneropenaeus chinensis*), Chinese mitten crab (*Eriocheir sinensis*), and snow crab (*Chionoecetes opilio*) were obtained through NCBI genome. The genomic information for the marbled crayfish (*P. virginalis*)^79^ was provided by Dr. Frank Lyko. Estimation of the proportion of duplicated genes (*P*_D_) was performed according to our previous study.^80^ After checking scores for Benchmarking Universal Single-Copy Orthologs (BUSCO), species with BUSCO <80% (i.e., *P. virginalis* and *C. opillo*; Table S3) were excluded from analysis as the incomplete genome annotation could lead to an over- and/or underestimation of the number of duplicated genes. Ecological information on decapods, including propagule size or invasiveness, was obtained from databases such as NEMESIS (https://invasions.si.edu/nemesis/) and AquaNIS (http://www.corpi.ku.lt/databases/index.php/aquanis/) and previous studies (summarized in Table S3). For other invertebrates, we referred to the data of our previous study.^81^ Phylogenetic generalized least squares analysis was then used to test the correlation between *P*_D_ and the propagule size while controlling for potential phylogenetic signals in them.

For each species and each gene, the longest transcript sequence was blasted against *D. melanogaster* using the BLASTp algorithm in BLAST v.2.11.0+,^80^ and a group of genes that top-hit to a given *D. melanogaster* gene was considered to belong to the gene family. For a given gene family, CAFE5^83^ was used to estimate the increase and decrease in the number of genes among species. CAFE5 models rates of gain and loss of genes with a birth–death distribution in all gene families. Then CAFE5 calculates the probability of observing the actual gene family size considering the given rate, and gene families that fall below the significance level are detected as rapidly evolving families. We first constructed a maximum likelihood (ML) tree with IQ-TREE^81^ using the concatenated protein sequences of single-copy orthologs. The sequences were aligned using MAFFT,^82^ and the gaps were trimmed by trimAl.^83^ The ML tree was then converted to an ultrametric tree using the chronopl function in the R package ape,^84^ with the estimated divergence time (409.3–536.3 mya) between Malacostraca and Insecta according to TimeTree,^85^ used as the divergence of crustaceans from the outgroup *D. melanogaster*.

### Quantification and statistical analysis

We have described the data analysis of this study in detail in the ‘Method details’ section. Results of statistical tests are presented as *P*-values in Figures 2 and 4, which are defined in the relevant figure legend, together with the name of the statistical test.

## Data Availability

• Supplementary data have been archived on figshare and are available at the URL listed in the key resources table. Sequenced read data have been deposited at the NCBI Sequence Read Archive and are publicly available as of the date of publication. The accession numbers are listed in the key resources table. • All original codes used for bioinformatic analyses has been deposited at github and are publicly available at the URL listed in the key resources table. • Any additional information required to reanalyze the data reported in this paper is available from the lead contact upon request.

## Supplemental information

Document S1. Figure S1 and Tables S1, S3, and S4

Table S2. KEGG and Reactome pathways enriched in the detected DEGs, related to Figure 2
