# Supplementary material for: Genomic adaptive potential to cold environments in the invasive red swamp crayfish
Source: iScience. 2023 Jul 3;26(8):107267. doi: 10.1016/j.isci.2023.107267 (PMC10371857; doi:10.1016/j.isci.2023.107267)
Supplement: Document S1. Figure S1 and Tables S1, S3, and S4 [file mmc1.pdf]

iScience, Volume *26*

Supplemental information

Genomic adaptive potential to cold environments in the invasive red swamp crayfish

Daiki X. Sato, Yuki Matsuda, Nisikawa Usio, Ryo Funayama, Keiko Nakayama, and Takashi Makino

Supplementary information

This file includes:

- Tables S1, S3, and S4
- Figure S1
- References

Table S1. **Sequence information of samples used in the present study, related to STAR Methods.**

Purpose	Species	Sample	#samples sequenced	#raw read pairs	#read pairs after quality control	Percentage (%)	Mapping rate (%)
Re-sequencing	*P. clarkii*	Atchafalaya	1	202,571,708	171,782,328	84.80	97.59
	P. zonangulus	Atchafalaya	1	247,045,666	194,339,204	78.67	91.54
	P. clarkii	SRR14457235[S1]	1	160,560,020	141,040,847	87.84	98.36
	P. alleni	SRR5115151[S2]	1	206,270,589	173,383,311	84.06	93.56
	P. fallax	SRR5115153[S2]	1	194,623,973	164,635,233	84.59	93.78
	P. virginalis	SRR5115141[S2]	1	193,514,202	167,456,117	86.53	93.67
Pool-sequencing	*P. clarkii*	New Orleans	6	178,643,626	145,600,414	81.50	97.50
	P. clarkii	Atchafalaya	8	159,143,107	132,707,222	83.39	97.38
	P. clarkii	Triunfo	7	197,658,499	165,946,614	83.96	98.06
	P. clarkii	Kamakura	8	176,803,657	147,172,607	83.24	95.95
	P. clarkii	Aomori	8	176,060,613	152,813,210	86.80	86.23
	P. clarkii	Sapporo	8	147,295,108	119,509,326	81.14	92.23
	P. clarkii	Okinawa	8	142,578,989	111,671,948	78.32	93.23
RNA-sequencing	*P. clarkii*	Sendai_Day0_1	1	11,932,427	11,708,413	98.12	92.58
	P. clarkii	Sendai_Day0_2	1	14,145,254	13,874,604	98.09	86.37
	P. clarkii	Sendai_Day0_3	1	12,002,925	11,787,048	98.20	90.27
	P. clarkii	Sendai_Day7_1	1	11,631,179	11,404,740	98.05	86.08
	P. clarkii	Sendai_Day7_2	1	11,026,231	10,813,188	98.07	83.11
	P. clarkii	Sendai_Day7_3	1	16,518,806	16,221,839	98.20	86.76
	P. clarkii	Sapporo_Day0_1	1	12,636,980	12,325,758	97.54	91.86
	P. clarkii	Sapporo_Day0_2	1	12,115,420	11,891,844	98.15	91.32
	P. clarkii	Sapporo_Day0_3	1	11,632,812	11,389,900	97.91	88.84
	P. clarkii	Sapporo_Day7_1	1	13,146,758	12,913,848	98.23	88.83
	P. clarkii	Sapporo_Day7_2	1	12,273,424	11,992,844	97.71	89.8
	P. clarkii	Sapporo_Day7_3	1	11,742,136	11,487,907	97.83	92.34
	P. clarkii	Sapporo_Day30_1	1	14,079,771	13,786,259	97.92	87.62

P. clarkii	Sapporo_Day30_2	1	10,271,630	10,055,561	97.90	85.01
P. clarkii	Sapporo_Day30_3	1	17,876,568	17,567,264	98.27	89.39

[S1] Xu et al. 2021

[S2] Gutekunst et al. 2018

Table S3. Proportion of duplicated genes and the ecological information of decapod species used in this study, related to Figure 4.

Species	Common name	#genes	#singletons	#duplicated genes	P_D	BUSCO (%)	Propagule size (cm)	NEMESIS	AquaNIS
Portunus trituberculatus	The Gazami crab	17,292	6,695	10,597	0.613	97.0	0.060[S3]	-	-
Homarus americanus	The American lobster	22,368	7,375	14,993	0.670	97.5	0.187[S4]	Invasive	Invasive
Penaeus chinensis	The Chinese white shrimp	20,076	6,883	13,193	0.657	95.6	0.029[S5]	-	-
Marsupenaeus japonicus	The Kuruma shrimp	22,301	7,175	15,126	0.678	98.1	0.033[S6]	-	-
Penaeus monodon	The giant tiger prawn	24,092	7,942	16,150	0.670	91.1	0.031[S7]	Invasive	-
Penaeus vannamei	The whiteleg shrimp	24,987	8,530	16,457	0.659	93.5	0.033[S8]	Invasive	-
Eriocheir sinensis	The Chinese mitten crab	19,615	6,184	13,431	0.685	97.9	0.051[S9]	Invasive	Invasive
Chinoecetes opilio	The snow crab	21,739	6,528	15,211	0.700	62.9	0.248[S10]	-	Invasive
Procambarus virginalis	The marbled crayfish	21,773	5,294	16,479	0.757	66.6	0.840[S11]	Invasive	Invasive
Procambarus clarkii	The red swamp crayfish	26,417	6,103	20,314	0.769	98.0	1.028[S12]	Invasive	Invasive

Table S4. The list of non-synonymous substitution sites significantly deviated in allele frequencies in the Sapporo population, related to Figure 5.

Chr	Position	Gene	Substitution	AF in US population			AF in Japanese population				SIFT	PROVEAN
				New Orleans	Atchafalaya	Triunfo	Aomori	Kamakura	Okinawa	Sapporo		
LG3	23664089	LOC123760978	V10F	0.218	0.213	0.229	0.260	0.267	0.608	0.886	**0.03**	-0.706
LG3	23668622	LOC123760978	A105T	0.539	0.356	0.302	0.591	0.342	0.260	0.905	0.17	0.007
LG3	23722086	LOC123758555	S309N	0.124	0.100	0.097	0.134	0.134	0.073	0.870	NA	0.000
LG3	23722279	LOC123758555	V245L	0.216	0.208	0.408	0.214	0.214	0.136	0.888	NA	0.000
LG3	23743176	LOC123760989	L123P	0.117	0.098	0.097	0.156	0.128	0.065	0.884	0.41	0.639
LG3	23800947	LOC123756530	F149L	0.384	0.545	0.519	0.311	0.320	0.172	0.921	**0.00**	-0.478
LG3	23803979	LOC123756530	A365V	0.465	0.269	0.216	0.228	0.213	0.119	0.912	0.13	-1.092
LG3	23976620	LOC123758616	E190Q	0.079	0.069	0.101	0.221	0.136	0.047	0.915	0.20	-1.104
LG3	24525914	LOC123761000	L175F	0.081	0.010	0.070	0.024	0.075	0.031	0.926	NA	**-4.000**
LG3	24983616	LOC123758696	T227S	0.090	0.036	0.085	0.036	0.090	0.038	0.920	**0.00**	-0.275
LG3	25622797	LOC123758717	Q16R	0.095	0.087	0.121	0.054	0.234	0.033	0.891	**0.00**	-0.196
LG3	25659785	LOC123758725	E367D	0.102	0.093	0.110	0.057	0.233	0.033	0.877	1.00	-0.303
LG8	27313522	LOC123752133	V408L	0.911	0.881	0.853	0.823	0.887	0.972	0.202	0.51	-0.368

LG8	27313551	LOC123752133	A398V	0.832	0.819	0.628	0.741	0.763	0.917	0.166	1.00	1.575
LG8	27589785	LOC123752289	Y157H	0.893	0.842	0.759	0.531	0.760	0.870	0.143	0.96	0.351
LG32	20442885	LOC123762164	E151D	0.386	0.658	0.533	0.320	0.270	0.216	0.872	NA	0.754
LG32	20447552	LOC123762165	S167P	0.092	0.086	0.108	0.189	0.089	0.057	0.819	NA	-0.063
LG32	20450656	LOC123762166	T176A	0.189	0.159	0.406	0.254	0.194	0.141	0.854	0.56	-0.170
LG32	20830534	LOC123762169	C312G	0.070	0.051	0.064	0.193	0.049	0.203	0.814	NA	-1.286

The bold text indicates significant effects of the substitutions as predicted by PROVEAN score (< –2.5) or SIFT score (< 0.05). SIFT could not be run for LOC123758555 and LOC123761000 owing to the lack of a sufficient number of orthologous sequences.

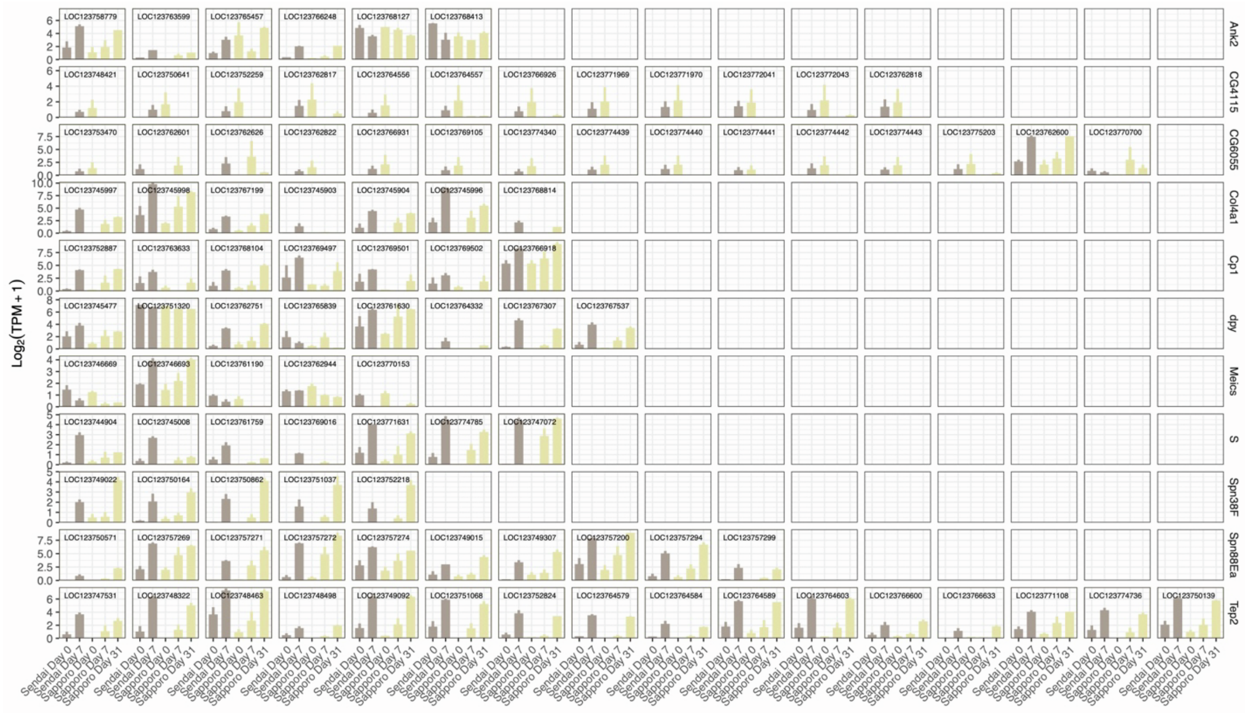

Figure S1. Expression profiles of rapidly expanded DEGs, related to Table 1.

Expression levels are shown in $\log_2$(TPM+1), and error bars represent standard errors (n = 3 for each condition).

References

[S1]. Xu, Z., Gao, T., Xu, Y., Li, X., Li, J., Lin, H., Yan, W., Pan, J., and Tang, J. (2021). A chromosome-level reference genome of red swamp crayfish Procambarus clarkii provides insights into the gene families regarding growth or development in crustaceans. Genomics *113*, 3274–3284. 10.1016/j.ygeno.2021.07.017.

[S2]. Gutekunst, J., Andriantsoa, R., Falckenhayn, C., Hanna, K., Stein, W., Rasamy, J., and Lyko, F. (2018). Clonal genome evolution and rapid invasive spread of the marbled crayfish. Nature Ecology and Evolution *2*, 567–573. 10.1038/s41559-018-0467-9.

[S3]. Wu, X., Cheng, Y., Zeng, C., Wang, C., and Yang, X. (2010). Reproductive performance and offspring quality of wild-caught and pond-reared swimming crab Portunus trituberculatus broodstock. Aquaculture *301*, 78–84. 10.1016/j.aquaculture.2010.01.016.

[S4]. Sibert, V., Ouellet, P., and Brêthes, J.-C. (2004). Changes in yolk total proteins and lipid components and embryonic growth rates during lobster (Homarus americanus) egg development under a simulated seasonal temperature cycle. Marine Biology *144*, 1075–1086.

[S5]. Heng, L., and Rui-yu, L. (1994). Comparative studies on the larval development of the penaeid shrimps,Penaeus Chinensis, P. merguiensis andP. penicillatus. Chin. J. Oceanol. Limnol. *12*, 295–307. 10.1007/BF02850489.

[S6]. Sato, T., Hamano, K., Sugaya, T., and Dan, S. (2017). Effects of maternal influences and timing of spawning on intraspecific variations in larval qualities of the Kuruma prawn Marsupenaeus japonicus. Mar. Biol. *164*, 70. 10.1007/s00227-017-3118-9.

[S7]. Silas, E.G., Muthu, M.S., Pijiai, N.N., and George, K.V. Larval development — Penaeus monodon Fabricius. CMFRI Bulletin *28*, 2–11.

[S8]. Kitani, H. (1986). Larval Development of the White Shrimp *Penaeus vannamei* BOONE Reared in the Laboratory and the Statistical Observation of its Naupliar Stages. Bulletin of the Japanese Society of Scientific Fisheries *52*, 1131–1139. 10.2331/suisan.52.1131.

[S9]. Chang, G., Wu, X., Cheng, Y., Zeng, C., and Yu, Z. (2017). Reproductive performance, offspring quality, proximate and fatty acid composition of normal and precocious Chinese mitten crab Eriocheir sinensis. Aquaculture *469*, 137–143. 10.1016/j.aquaculture.2016.11.025.

[S10]. Adams, A.E. (1979). The life history of the snow crab, Chionoecetes opilio : a literature review. Alaska sea grant report *78–13*.

[S11]. Vogt, G., Tolley, L., and Scholtz, G. (2004). Life stages and reproductive components of the Marmorkrebs (marbled crayfish), the first parthenogenetic decapod crustacean. J. Morphol. *261*, 286–311. 10.1002/jmor.10250.

[S12]. Amer, M.A., M., E.-S.A.A., Al-Damhougy, K.A., Zaakouk, S.A., and Ghanem, M.H. Changes in uropod setae during molting of the freshwater crayfish, Procambarus clarkii. International Journal of Advanced Research *3*, 360–367.
